# Operational research to inform post-validation surveillance of lymphatic filariasis in Tonga study protocol: History of lymphatic filariasis elimination, rational, objectives, and design

**DOI:** 10.1371/journal.pone.0307331

**Published:** 2024-08-20

**Authors:** Harriet Lawford, ‘Ofa Tukia, Joseph Takai, Sarah Sheridan, Colleen L. Lau

**Affiliations:** 1 UQ Centre for Clinical Research, The University of Queensland, Brisbane, QLD, Australia; 2 Public Health Division, Ministry of Health, Nuku’alofa, Tonga; 3 National Centre for Immunisation Research and Surveillance of Vaccine Preventable Diseases, Sydney, NSW, Australia; Public Library of Science, UNITED KINGDOM OF GREAT BRITAIN AND NORTHERN IRELAND

## Abstract

**Background:**

Lymphatic filariasis (LF), a mosquito-borne helminth infection, is an important cause of chronic disability globally. The World Health Organization has validated eight Pacific Island countries as having eliminated lymphatic filariasis (LF) as a public health problem, but there are limited data to support an evidence-based approach to post-validation surveillance (PVS). Tonga was validated as having eliminated LF in 2017 but no surveillance has been conducted since 2015. This paper describes a protocol for an operational research project investigating different PVS methods in Tonga to provide an evidence base for national and regional PVS strategies.

**Methods:**

Programmatic baseline surveys and Transmission Assessment Surveys conducted between 2000–2015 were reviewed to identify historically ‘high-risk’ and ‘low-risk’ schools and communities. ‘High-risk’ were those with LF antigen (Ag)-positive individuals recorded in more than one survey, whilst ‘low-risk’ were those with no recorded Ag-positives. The outcome measure for ongoing LF transmission will be Ag-positivity, diagnosed using Alere™ Filariasis Test Strips. A targeted study will be conducted in May-July 2024 including: (i) high and low-risk schools and communities, (ii) boarding schools, and (iii) patients attending a chronic-disease clinic. We estimate a total sample size of 2,010 participants.

**Conclusions:**

Our methodology for targeted surveillance of suspected ‘high-risk’ populations using historical survey data can be adopted by countries when designing their PVS strategies. The results of this study will allow us to understand the current status of LF in Tonga and will be used to develop the next phase of activities.

## Introduction

Lymphatic filariasis (LF) is a mosquito-borne parasitic infection caused by three species of filarial worms (*Wuchereria bancrofti*, *Brugia malayi*, or *Brugia timori*) [1]. In Tonga, diurnally sub-periodic *W*. *bancrofti* is the dominant species of parasite causing LF [2], which is transmitted primarily by *Aedes tongae* and *Ae*. *tabu mosquitoes* [3]. Both vectors are diurnal feeders that feed indoors and outdoors, and are container breeders with habitats that include tree and coconut holes, leaf axils, and artificial containers [4].

The Global Programme to Eliminate LF (GPELF) is one the largest public health programs in the world [5]. GPELF was launched by the World Health Organization (WHO) in 2000 with the aim to i) interrupt transmission through mass drug administration (MDA) of anthelminthic medicines, and ii) control morbidity of affected populations by 2020 [6]. New milestones and targets beyond 2020 have been developed; the *WHO Neglected Tropical Diseases Roadmap 2030* [7] proposes that all countries complete their MDA programmes, implement post-MDA or post-validation surveillance, and have implemented a minimum package of care for LF morbidity by 2030 [8]. MDA aims to reduce the prevalence of infections to a level where transmission is thought to be no longer sustainable [9], with the elimination criteria for *Aedes* vector areas set at <1.0% antigen (Ag) prevalence in 6-7-year-old children.

Many countries in the Pacific region have achieved validation of LF elimination; however, there is a limited evidence base to inform the development of effective and efficient PVS strategies. Operational research is required to determine effective sampling strategies to confirm the presence or absence of LF transmission post-validation, to identify any ongoing transmission in a cost-effective and timely manner, and to integrate surveillance into other public health programs to ensure long-term sustainability.

### History of LF elimination activities in Tonga, 1925 to 2017

**Fig 1:**
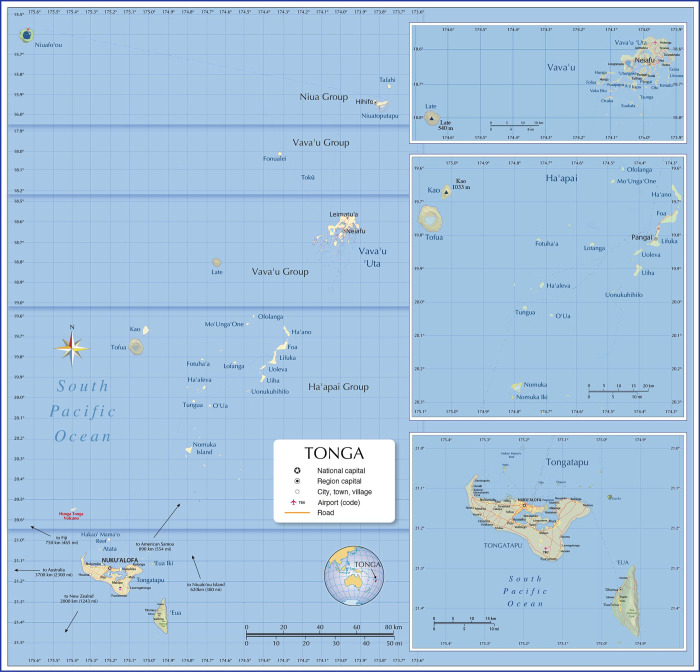
Map of the Kingdom of Tonga. Source: Nations Online Project (https://www.nationsonline.org/oneworld/map/tonga-map.htm).

The Kingdom of Tonga is an archipelago of over 170 islands (Fig 1). Historical surveys of LF prevalence in Tonga recorded microfilaria (Mf) prevalence ranging from 13.5% in Tongatapu (1925) to 71.0% in Niuatoputapu (1970), and nationwide Mf prevalence of 17.4% in 1976 [10]. In 1977, MDA of diethylcarbamazine (DEC) (one dose/month for 12 months) was implemented, following which nationwide surveys in 1979, 1983/4, and 1998/9 recorded Mf prevalence of 1.0%, 0.4%, and 0.63%, respectively, suggesting ongoing residual transmission [10].

The Pacific Programme for the Elimination of LF (PacELF) was launched in 2000, following which the Ministry of Health (MOH) in Tonga established the National Programme to Eliminate LF (NPELF) which aimed to (i) achieve 100% geographic coverage for MDA by the year 2001, (ii) implement five effective rounds of MDA throughout the country, and (iii) achieve interruption of transmission by 2005 [10]. Each division/island group was designated as an implementation unit (IU), giving a total of five IUs: ‘Eua, Ha’apai, Ongo Niua, Tongatapu, and Vava’u.

A baseline survey (A Survey) of convenience sampling of adults (n = 4,002) at sentinel sites was conducted in 1999–2000 (prior to the first round of MDA) and found an Ag prevalence of 2.7% (n = 108 positive), following which three rounds of MDA (DEC at 6 mg/kg body weight and one tablet of albendazole at 400 mg) were distributed throughout the country between 2001–2003 [11]. Following the third round of MDA, a mid-term evaluation (B Survey) of adults (n = 3,294) in sentinel sites was implemented in 2003–2004, which recorded an Ag prevalence of 2.5% (n = 81 positive) [11]. Two further MDA rounds were conducted in 2004 and 2005. In 2006, a pre-stop MDA survey (C Survey) was implemented in all sites/villages in Ongo Niuas (where a high baseline Ag prevalence was recorded) and 4–6 sites in the remaining IUs. Overall, 2,927 participants were surveyed and 11 were Ag-positive, giving an overall Ag prevalence of 0.4% [11]. Five positive cases were identified in Niuatoputapu district, Ongo Niua, five in Ha’apai, and one in ‘Eua. Following the C Survey, a further round of MDA was distributed in 2006 in Niuatoputapu district only.

To determine whether MDA could be stopped, a stop MDA survey (Transmission Assessment Survey [TAS]-1/D Survey) was conducted in 2007 among 2,391 First Grade children (5–6 years old) in all IUs, with no children testing positive. In addition, dried blood spots (DBS) were collected from 797/801 children from ‘Eua, Ha’apai, and Vava’u for antifilarial antibody (Ab) testing with the Filariasis ELISA (Joseph *et al*, 2011 [12]). An overall *Bm14* Ab prevalence of 6.3% was reported among these children, though there were no Ag-positive cases, suggesting that LF transmission remained successfully interrupted [12]. Of note, the five individuals from Niuatoputapu district, Ongo Niua, who tested positive in the 2006 C Survey were retested in 2007, and three of the five remained Ag-positive [11]. No Mf testing was conducted on these individuals.

**Fig 2:**
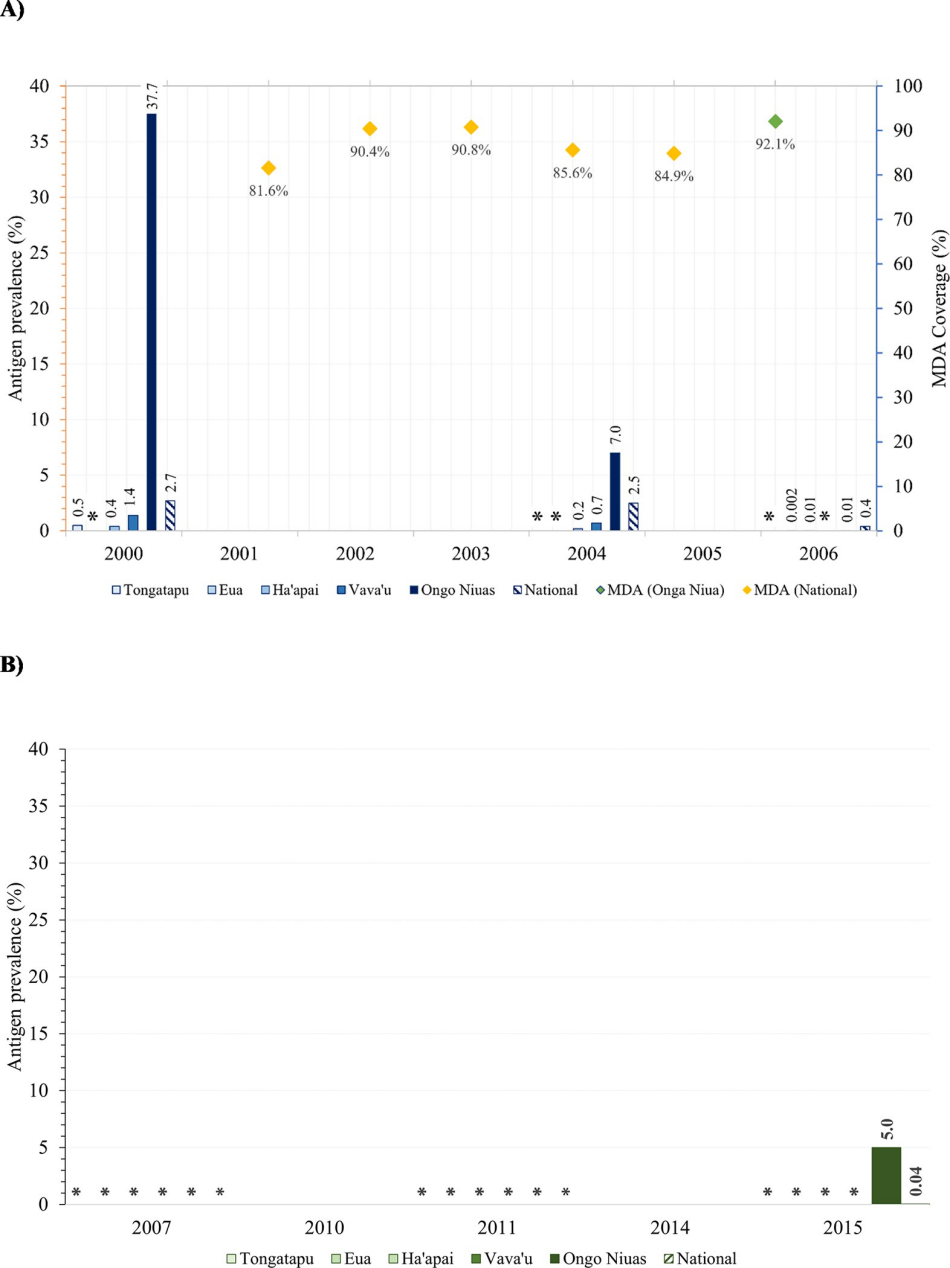
Programmatic baseline surveys (A) and Transmission Assessment Surveys (B) indicating antigen prevalence and mass drug administration coverage between 2000–2015, Tonga. Asterisks indicate an antigen prevalence of 0% (no Ag-positives were found).

TAS-2 was conducted in all IUs in 2011 and returned no positive samples among 2,451 children. However, a survey by Chu *et al*. (2014), which aimed to simultaneously assess the impact of MDAs on LF and STH by incorporating STH testing into TAS-2, reported 7/2,434 Ag-positive students from six schools in the IUs Tongatapu (n = 3), Ha’apai (n = 2), and Ongo Niua (n = 2), giving an overall Ag prevalence of 0.3% [13]. A final TAS (TAS-3) was conducted in 2015 among 2,806 children from all five divisions; one child tested positive in Ongo Niua, giving an overall Ag prevalence 0.04%, suggesting continued interruption of LF transmission in Tonga. In 2017, Tonga successfully obtained WHO’s validation of elimination of LF as a public health problem [10]. Please refer to S1 Table for details of previous LF surveys in Tonga and Fig 2 for the estimated Ag prevalence and MDA coverage in the years leading to LF elimination in Tonga.

### Study rationale

The identification of seven positive children in the 2011 TAS-2, and one positive child in the 2015 TAS-3, suggests that there may be persistent localised areas of LF transmission in some communities in Tonga despite five rounds of nationwide MDA (and six rounds of MDA in Niuatongatapu district, Ongo Niua). There has been no community surveillance of LF since 2015, thus transmission may have been re-established but not yet been identified. This study will be the first to determine whether LF transmission is still occurring in Tonga five-years post-validation of LF elimination, and if transmission is found, will enable early identification of LF to allow a timely response to prevent widespread recrudescence of LF in Tonga. Further, we will investigate different PVS methodologies to determine the most efficient method to identity residual LF transmission in Tonga, and thereby provide an evidence-base for national and regional PVS strategies.

In WHO’s Global Programme to Eliminate Lymphatic Filariasis, school-based TAS of 6-7-year-old school children are currently used to determine whether Ag prevalence has dropped below the critical threshold of 1.0% to signify LF elimination. However, rather than conducting a study to estimate Ag prevalence, we have chosen to adopt a targeted surveillance approach that aims to sample areas/people with the highest probability of LF infection to determine the presence or absence of disease. We believe that there are several advantages to conducting targeted surveillance for PVS. Firstly, the justification for conducting TAS among 6-7-year-olds is that young children should be protected from LF infection (and thus have low or zero Ag prevalence) if previous MDA rounds were successful. Furthermore, antigenemia detected in older children and adults may be due to infections pre-dating MDA [14]. However, previous surveys have shown that TAS-like sampling of children performs poorly for detecting microfoci of ongoing transmission, with significant persistence of LF reported in adults despite areas having met elimination targets in school-based TAS [15]. Studies have also reported significantly higher Ag prevalence in adults compared to children [16], and in community surveys (especially true for people >30 years) [17]. Therefore, we have chosen to test older aged school children (in the last two Grades/Forms of primary/high school) as well as community members to determine whether this increases the likelihood of detecting Ag-positives. We have also chosen to test children in the last two years of boarding school, as this provides a unique opportunity to access older, high-school aged children who (i) may have a higher Ag prevalence than 6-7-year-olds, and (ii) are from remote island communities that can be hard to access and may otherwise be missed.

We will also conduct a clinic-based survey. We hypothesise that individuals suffering from chronic diseases may be less likely to have taken MDA in previous rounds due to serious illness or co-morbidities, or concerns about taking MDA in addition to their regular medications, and may therefore more likely to have untreated LF infections. These ‘never treated’ individuals could potentially be acting as reservoirs of infection in communities, leading to sustained LF transmission [18–20]. Integrating LF testing into pre-established screening activities for patients attending chronic disease clinics is a less resource intensive and convenient means to test this potentially high-risk population.

Lastly, evidence suggests that anti-filarial Ab markers could be more sensitive measures of transmission than Ag in low prevalence settings [21], and Ab prevalence may indicate pre-antigenemic LF infection and ongoing transmission or resurgence, thereby allowing a timelier response compared to testing for Ag alone [21–23]. This study will concurrently measure seroprevalence of LF Ag and Abs, thereby providing an opportunity to determine the utility of Ab as a marker of LF transmission or resurgence, and assess the sensitivity of different diagnostic tools in PVS settings.

### Aim and objectives

The aim of this study is to investigate different approaches for PVS of LF and provide an evidence base for developing ongoing PVS strategies in Tonga and regionally. The primary objective is to determine if there is any evidence of ongoing transmission of LF in Tonga post-validation of elimination of LF as a public health problem. In addition, we will:

1. estimate the prevalence of Mf and Ag in communities with ongoing transmission.
2. describe the demographic characteristics and geographical distribution of Ag-positive individuals (if found).
3. investigate whether anti-filarial Abs (*Wb123* Ab, *Bm14* Ab, *Bm33* Ab) provide an earlier signal of ongoing transmission compared to Ag.
4. determine whether seroprevalence of Abs vary between populations with recently recorded LF exposure, and populations with no recorded history of LF exposure.

## Methods

### Study setting

To confirm the presence/absence of LF, this study will be conducted in four different settings: primary schools (‘low-risk’ and ‘high-risk’), high schools (including boarding schools), communities (‘low-risk’ and ‘high-risk’), and a diabetes outpatient clinic based at the national hospital (Table 1).

**Table 1:** Study setting and target sample size.

Setting	Reason for inclusion	Participants to be tested*	Total sites	Target sample size
‘High-risk’ primary schools	Ag-positive schools in TAS 2 (n = 6) and school located in community with high Ag-prevalence in B-Survey (n = 1)	Grades 5–6	7	385
‘Low-risk’ primary schools	No Ag-positive schools identified in TAS 1, 2, or 3, and no Ag-positives recorded in community-based C survey	Grades 5–6	4	220
Boarding school	Students boarding from outer islands	Forms 6–7	4	200
High school	School located in area of historically high Ag prevalence	Forms 6–7	1	50
‘High-risk’ communities	Households in the villages where Ag-positive school children were identified in TAS 2 (n = 6) and community with high Ag-prevalence in B-Survey (n = 1)	All consenting household members aged ≥5 years	7	525
‘Low-risk’ communities	Communities that recorded no Ag-positive cases in the 2006 community-based C survey	All consenting household members aged ≥5 years	4	300
Diabetes clinic	Recruitment of chronically ill patients who may have been missed in previous MDA rounds	Patients presenting to the clinic	1	200
**TOTAL**			**28**	**1880^**

*In primary schools with low attendance numbers, Grades 3–4 and Forms 1 and 2 will also be included, and in boarding/high schools with low attendance numbers, Forms 4–5 and Technical and Vocational Education students will also be included

^This number may increase if Ag-positive participants are identified from the school and clinic-based surveys, and their household members are tested

### Identification of study sites

Programmatic baseline surveys and TAS conducted between 2000–2015 were reviewed to identify ‘high-risk’ and ‘low-risk’ locations. ‘Low-risk’ locations will be used as reference populations against which LF Ag and Ab seroprevalence from the targeted, high-risk schools and villages can be compared. The six primary schools that recorded Ag-positive children in the 2011 TAS were considered ‘high-risk’ primary schools, and the communities in which the schools are located were considered ‘high-risk’ communities. An additional primary school was selected as it was in a community that recorded high Ag prevalence in the 2003 B Survey. Four primary schools that recorded no Ag-positive children in 2011 TAS were considered low-risk reference schools; these schools were from ‘low-risk’ communities that also recorded no positive cases in the 2006 community-based C survey. Following consultation with the MOH, boarding schools that were believed to have a high proportion of students boarding from outer islands were selected. Lastly, following consultation with MOH, the diabetes clinic at Vaiola Hospital in Nuku’alofa was selected for the clinic-based component of the study due to large number of patients who could be recruited.

### Participant selection and target sample size

At selected schools, all students in Grades 5–6 of primary school, and Forms 6–7 of high school, will be enrolled in the study. Based on estimated student enrolment numbers from the Tonga Statistics Department [24], we estimate approximately 55 students to be enrolled per primary school (25 students in Grade 5 and 30 students in Grade 6), and 50 students per high school (25 students in Form 6 and 25 students in Form 7). In schools with low enrolment numbers, eligible grades in primary school will be expanded to include Grades 3–4 and Forms 1–2, and in high schools will be expanded to include Forms 4–5 and Technical and Vocational Education (TVET) students. If any Ag-positive students are identified, they will be followed-up at home and all household members ≥5 years old will be offered testing.

For the community survey, a list of households in the selected villages will be obtained from the Tonga Statistics Department. All households will be assigned a number from one to the total number of households per village. The total number of households in each village will be entered into a Random Number Generator. Fifteen unique random numbers between one and the total number of households per village will be generated. We will survey the households represented by these numbers. An additional five households per village will be selected in case any of the selected households are uninhabited or the inhabitants cannot be reached. Based on Tonga’s Population and Housing Census 2021 [25], we estimate five participants per household and 75 participants per community.

For the clinic-based survey, all patients attending the diabetes clinic at Vaiola Hospital, Nuku’alofa, during the survey weeks will be invited to participate. The target sample size will be 200 patients (if no Ag-positives are detected, this provides an upper 95% CI of 1.5%). If any Ag-positive patients are identified, they will be followed-up at home and all household members ≥5 years old will be tested.

### Ethics

The study has been approved by the Tongan National Health Ethics and Research Committee of the Tongan Ministry of Health (Ref#20240129) and ratified by The University of Queensland’s Human Research Ethics Committee (Project#2024/HE000493).

### Eligibility criteria and consent process

For the school-based survey, all children in the selected Grades or Forms will be invited to participate. Approximately one week prior to the survey, school principals will be informed about the survey by a team member. Principals or teachers will be asked to distribute participant information sheets and consent forms to all children in the selected Grades or Forms and children will be asked to give these forms to their parents to sign. On the day of the survey, team members will collect signed consent forms and enrol consented students into the study.

For the community-based survey, all household members aged ≥5 years will be eligible to participate. A person will be considered a household member if they slept at the house the previous night or identify the house as their primary residence. Upon arrival at the household, a standard participant information sheet and consent form will be administered to all study participants. A team member will explain the purposes of the study and seek written consent (parental or guardian consent in the case of a children <18 years of age) to participate in the study. The total number of household members including those who are absent, will be recorded.

At the diabetes clinic, patients will be recruited whilst they are in the waiting area prior to their scheduled appointment. A team member will explain the purposes of the study and seek written consent (parental or guardian consent in the case of a children <18 years of age) to participate in the study.

All participants will be advised that they can revoke their consent at any time without any prejudice.

### Questionnaires

Standardized electronic questionnaires will be administered using EpiCollect5 [26]. Questionnaires will be used to collect demographic information (including age, sex, country/place of birth, village of residence, occupation) and travel history in the past 12 months (both international and within Tonga). At selected households, schools, and chronic disease clinic, an environmental assessment will be conducted by trained Environmental Health Officers as part of their routine inspections. These inspections will include an assessment of the materials of the household/school/clinic structure, water sourcing and storage, and potential vector breeding sites. GPS coordinates of each enrolled household will be recorded.

### Blood collection and laboratory testing

For each enrolled participant, at least 300μL of blood will be collected by finger prick into heparin-coated BD Microtainer® Blood Collection Tubes. Alere™ Filariasis Test Strips (FTS) (Abbott, Scarborough, ME) will be used to detect LF antigen. For any Ag-positive samples, Mf slides will be prepared using methods described previously in the literature [27]. DBS will be collected from all individuals (irrespective of Ag positivity) for Multiplex Bead Assays (MBA) to detect anti-filarial Abs using methods described previously in the literature [21].

### Sample and data linkage

To enable linkage of demographic variables, environmental variables, and FTS/DBS results collected in EpiCollect5, each participant will be given a unique identifying number that will be printed as QR code stickers and attached to consent forms, questionnaires, blood collection tubes, FTS, slides, and dried blood spots.

### Data analysis plan

The primary outcome measure to signal the presence of LF in Tonga will be a positive Ag test. Crude Ag and Ab prevalence with 95% confidence intervals (CI) will be estimated using binomial exact methods. Seroprevalence of LF Ab will be estimated by measuring IgG responses using MBA with Ag-specific cut-off values (measured by Median Fluorescence Intensity [MFI-bg]) used to determine seropositivity. Ag and Ab prevalence estimates will be adjusted for survey design and sex distribution for the school-based surveys, and adjusted for survey design, sex, and age distribution for the community-based survey based on Tonga’s Population and Housing Census 2021 [25].

Differences in demographic characteristics between Ag/Ab positive individuals will be described using mean ± standard deviation (SD), median [interquartile range], or number (%), and tested using Student’s t -test or Mann–Whitney U test for continuous data and Pearson’s chi-squared test of independence or Fisher’s exact test for categorical data. Logistic regression will be used to assess any associations between demographic variables and Ag/Ab positivity. Any variables with p <0.2 on univariate analyses will be tested using multivariable logistic regression. Variables will be assessed using variation inflation factor <5 to assess for potential collinearity, and final models will be selected using backward elimination, wherein variables are sequentially removed from the multivariable models to arrive at the most parsimonious models, in which variables with p <0.05 will be retained.

If possible, the sensitivity of Ag vs Ab to detect LF transmission in the post-validation period will be determined as the percentage of individuals with a positive FTS test among those testing Ab positive using MBA. A Kappa statistic will be performed to estimate the strength of agreement between FTS and Abs. The weights for agreement will be ranged as follows: *k* < 0.00 to indicate no agreement, *k* 0.00–0.20 poor agreement, *k* 0.21–0.40 fair agreement, *k* 0.41–0.60 moderate agreement, *k* 0.61–0.80 substantial agreement, and *k* 0.81–1.00 almost perfect agreement. Lastly, seroprevalence estimates and mean MFI-bg values will be compared between communities with a history of high LF transmission and ‘low-risk’ reference communities for significant differences.

### Recruitment

Recruitment of participants began on the 8^th^ May 2024 and is anticipated to be completed by the 31^st^ July 2024.

## Discussion

This study is a unique opportunity to test the effectiveness of PVS strategies in the Pacific Island setting, which may provide evidence for strategies that will be applicable to countries and territories with similar contexts. The methodology adopted in this study will provide an evidence base to develop PVS of LF in Tonga and other Pacific Island countries that are at a similar stage of LF elimination. In this protocol, we propose adopting a targeted sampling approach of ‘high-risk’ individuals from areas with historically high Ag prevalence based on survey data, and ‘low-risk’ individuals from areas where no Ag-positive individuals were identified in previous surveys who can serve as a reference population. A targeted sampling approach has the advantage of being a cost-effective means to detect the presence or absence of LF.

The results of this survey will allow us to understand the current status of LF in Tonga. This information will be used to develop the next phase of activities and the development of an appropriate strategic response to the following scenarios:

- If no Ag-positive individuals are identified in areas where LF transmission is most likely to be found, we can be highly confident that LF has been eliminated as a public health problem in Tonga;
- If there is low Ag prevalence, our results will be useful to inform a more extensive survey, and ongoing PVS strategy for Tonga; and
- If high prevalence of Ag-positive, or any Mf-positive individuals are identified, more intensive surveillance and targeted MDA could be considered.

Depending on the study’s findings, other potential strategies for ongoing surveillance of LF could be considered, including opportunistic testing of blood samples from blood donors, antenatal clinics, pre-employment medicals, military recruits, and/or routine blood tests.

Lastly, we propose collecting DBS for MBA that will provide opportunities to measure the seroprevalence of anti-filarial Abs. MBA analysis will facilitate an assessment of the utility of Ab serosurveillance to signal LF transmission or resurgence, thereby providing evidence for PVS strategies that will be applicable to similar contexts in the region. Estimating Ab seroprevalence will provide opportunities to (i) further investigate the interpretation of Ab signals; and (ii) assess the sensitivity of Ab vs Ag to signal ongoing LF transmission, thereby providing evidence for PVS strategies that will be applicable to similar contexts in the region.

## Supporting information

S1 Table Detailed timeline of milestones towards LF elimination in Tonga. (DOCX)
