# Supplementary material for: Operational research to inform post-validation surveillance of lymphatic filariasis in Tonga study protocol: History of lymphatic filariasis elimination, rational, objectives, and design
Source: PLoS One. 2024 Aug 20;19(8):e0307331. doi: 10.1371/journal.pone.0307331 (PMC11335152; doi:10.1371/journal.pone.0307331)
Supplement: S1 Table — (DOCX) [file pone.0307331.s001.docx]

**S1 Table. Detailed timeline of milestones towards LF elimination in Tonga**

| **Event (year)** | **MDA** | | **Sample** | | | **Location Surveyed** | | | | | |
| --- | --- | --- | --- | --- | --- | --- | --- | --- | --- | --- | --- |
|  | **Location (Island Group)** | **Treatment coverage, n (%)** | **Sampling strategy** | **Total tested (n)** | **National Ag prevalence, n (%)** | **Island Group** | **District** | **Village** | **Total tested, n** | **Total Ag-positive, n (%)** | **Total Mf-positive n (%)** |
| A Baseline survey (1999-2000) |  |  | Convenience sampling of LF Ag prevalence in villages/villages with high endemicity (sentinel sites) in all 5 divisions. | 4,002 | 109 (2.7) | Tongatapu |  |  | 1934 | 10 (0.5) | NT |
|  |  |  |  |  |  | Vava'u |  |  | 1065 | 15 (1.4) | NT |
|  |  |  |  |  |  | Ongo Niua |  |  | 215 | 81 (37.7) | NT |
|  |  |  |  |  |  | Ha'apai |  |  | 688 | 3 (0.4) | NT |
|  |  |  |  |  |  | 'Eua |  |  | 100 | 0 | NT |
| MDA 1 (2001) | National | 79,969 (81.6) |  |  |  |  |  |  |  |  |  |
| MDA 2 (2002) | National | 82,023 (90.4) |  |  |  |  |  |  |  |  |  |
| MDA 3 (2003) | National | 88,752 (90.8) |  |  |  |  |  |  |  |  |  |
| B Mid-term Survey (2003-4) |  |  | Convenience sampling of LF Ag prevalence in villages/villages with high endemicity (sentinel sites) in 4/5 divisions. Two surveys conducted in 2003 (targeted Tongatapu, Vava'u and Niuatoputapu, and Ha'apai) and 2004 ('O'ua Island, Ha'apai division). | 3,294 | 81 (2.5) | Tongatapu |  |  | 533 | 0 | NT |
|  |  |  |  |  |  | Vava'u |  |  | 1043 | 7 (0.7) | NT |
|  |  |  |  |  |  | Ongo Niua | Niuatoputapu |  | 860 | 60 (7.0) | NT |
|  |  |  |  |  |  |  | Niuafo’ou |  | ND | ND | NT |
|  |  |  |  |  |  | Ha'apai |  |  | 858 | 14 | NT |
|  |  |  |  |  |  | ‘Eua |  |  | ND | ND | NT |
| MDA 4 (2004) | National | 83,719 (85.6) |  |  |  |  |  |  |  |  |  |
| MDA 5 (2005) | National | 93,218 (84.9) |  |  |  |  |  |  |  |  |  |
| C Pre-stop MDA Survey (2006) |  |  | Cross-sectional survey of all 5 divisions. | 2,927 | 11 (0.4) | Tongatapu | Tatakamotonga | Tatakamotonga | 146 | 0 | 0 |
|  |  |  |  |  |  |  | Vaini | Veitongo | 130 | 0 | 0 |
|  |  |  |  |  |  |  | Kolomotu’a | Sia’atoutai | 95 | 0 | 0 |
|  |  |  |  |  |  |  |  | Kolomotu’a | 120 | 0 | 0 |
|  |  |  |  |  |  | Vava'u | Hihifo | Vaimalo | 60 | 0 | 0 |
|  |  |  |  |  |  |  | Motu | Kapa | 42 | 0 | 0 |
|  |  |  |  |  |  |  |  | Nuapapu | 48 | 0 | 0 |
|  |  |  |  |  |  |  | Neiafu | Toula | 164 | 0 | 0 |
|  |  |  |  |  |  |  |  | Neiafutahi | 137 | 0 | 0 |
|  |  |  |  |  |  | Ongo Niua | Niuatoputapu | Hihifo | 240 | 2 (0.3) | 1 (0.4) |
|  |  |  |  |  |  |  |  | Falehau | 185 | 1 (0.2) | 1 (0.5) |
|  |  |  |  |  |  |  |  | Vaipoa | 173 | 2 (0.3) | 0 |
|  |  |  |  |  |  |  |  | Tafahi | 32 | 0 | 0 |
|  |  |  |  |  |  |  | Niuafo’ou | Mu’a | 24 | 0 | 0 |
|  |  |  |  |  |  |  |  | Sata’ata | 86 | 0 | 0 |
|  |  |  |  |  |  |  |  | Petani | 66 | 0 | 0 |
|  |  |  |  |  |  |  |  | Fata’ulua | 52 | 0 | 0 |
|  |  |  |  |  |  |  |  | ‘Esia | 87 | 0 | 0 |
|  |  |  |  |  |  |  |  | Mata’aho | 24 | 0 | 0 |
|  |  |  |  |  |  |  |  | Kolofo’ou | 82 | 0 | 0 |
|  |  |  |  |  |  |  |  | Tongamama’o | 42 | 0 | 0 |
|  |  |  |  |  |  | Ha'apai | Ha’ano | Fakakakai | 107 | 2 (0.4) | 0 |
|  |  |  |  |  |  |  | Foa | Fotua | 75 | 3 (0.6) | 0 |
|  |  |  |  |  |  |  |  | Faleoa | 111 | 0 | 0 |
|  |  |  |  |  |  |  | Pangai | Holopeka | 65 | 0 | 0 |
|  |  |  |  |  |  |  | Pangai | Koulo | 53 | 0 | 0 |
|  |  |  |  |  |  |  | Lulunga | ‘O’ua | 57 | 0 | 0 |
|  |  |  |  |  |  | ‘Eua | 'Eua Fo'ou | Angaha | 107 | 1 (0.2) | 0 |
|  |  |  |  |  |  |  |  | Mata’aho | 99 | 0 | 0 |
|  |  |  |  |  |  |  |  | Fata’ulua | 95 | 0 | 0 |
|  |  |  |  |  |  |  | 'Eua Motu’a | Ha’atu’a | 123 | 0 | 0 |
| MDA 6 (2006) | Niuatoputapu (Ongo Niuas) | 923 (92.1) |  |  |  |  |  |  |  |  |  |
| D TAS1 / Stop MDA Survey (2007) |  |  | School-based survey of all Grade 1 children (approximately 6 years old). | 2,391 | 0 (0.0) | Tongatapu |  |  | 1509 | 0 | 0 |
|  |  |  |  |  |  | Vava'u |  |  | 413 | 0 | 0 |
|  |  |  |  |  |  | Ongo Niua |  |  | 81 | 0 | 0 |
|  |  |  |  |  |  | Ha'apai |  |  | 219 | 0 | 0 |
|  |  |  |  |  |  | 'Eua |  |  | 169 | 0 | 0 |
|  |  |  | Five positive cases from Ongo Niua who tested positive in C Survey retested. | 5 | 3 (60.0) | Ongo Niua | Niuatoputapu | Hihifo | 2 | 1 (50.0) | NT |
|  |  |  |  |  |  |  |  | Falehau | 1 | 1 (100.0) | NT |
|  |  |  |  |  |  |  |  | Vaipoa | 2 | 1 (50.0) | NT |
| TAS2 / Post-MDA Surveillance (2011) and results from Chu *et al* (2014) [13] |  |  | School-based survey of all Grade 1 children (approximately 6 years old). | 2,468 | 7 (0.3) | Tongatapu (West) | Kolovai | 'Atata | 1 | 0 | 0 |
|  |  |  |  |  |  |  |  | Fahefa | 13 | 0 | 0 |
|  |  |  |  |  |  |  |  | Kanokupolu | 10 | 0 | 0 |
|  |  |  |  |  |  |  |  | Kolovai | 39 | 0 | 0 |
|  |  |  |  |  |  |  | Nukunuku | Fatai | 11 | 0 | 0 |
|  |  |  |  |  |  |  |  | Ha'alalo | 19 | 0 | 0 |
|  |  |  |  |  |  |  |  | 'Utualu | 19 | 1 (5.2) | 0 |
|  |  |  |  |  |  |  |  | Vaotu'u | 14 | 2 (14.3) | 0 |
|  |  |  |  |  |  |  |  | Nukunuku | 19 | 0 | 0 |
|  |  |  |  |  |  |  |  | Matahau | 11 | 0 | 0 |
|  |  |  |  |  |  |  |  | Houma | 48 | 0 | 0 |
|  |  |  |  |  |  | Tongatapu (Central) | Kolofo’ou | Kolofo’ou | 440 | 0 | 0 |
|  |  |  |  |  |  |  |  | Popua | 24 | 0 | 0 |
|  |  |  |  |  |  |  |  | Ma’ufanga | 73 | 0 | 0 |
|  |  |  |  |  |  |  | Kolomotu’a | Havelu | 68 | 0 | 0 |
|  |  |  |  |  |  |  |  | Kolomotu’a | 179 | 0 | 0 |
|  |  |  |  |  |  |  |  | Sia’atoutai | 11 | 0 | 0 |
|  |  |  |  |  |  |  |  | Tofoa | 27 | 0 | 0 |
|  |  |  |  |  |  |  |  | Puke | 15 | 0 | 0 |
|  |  |  |  |  |  |  |  | Hofoa | 18 | 0 | 0 |
|  |  |  |  |  |  | Tongatapu (East) | Lapaha | ‘Eueiki | 6 | 0 | 0 |
|  |  |  |  |  |  |  |  | Afa | 18 | 0 | 0 |
|  |  |  |  |  |  |  |  | Hoi | 13 | 0 | 0 |
|  |  |  |  |  |  |  |  | Kolonga | 22 | 0 | 0 |
|  |  |  |  |  |  |  |  | Lapaha | 44 | 0 | 0 |
|  |  |  |  |  |  |  |  | Talafo’ou | 13 | 0 | 0 |
|  |  |  |  |  |  |  |  | Nukuleka | 10 | 0 | 0 |
|  |  |  |  |  |  |  |  | Niutoua | 13 | 0 | 0 |
|  |  |  |  |  |  |  |  | Navutoka | 16 | 0 | 0 |
|  |  |  |  |  |  |  | Tatakamotonga | Fatumu | 14 | 0 | 0 |
|  |  |  |  |  |  |  |  | Fua’amotu | 17 | 0 | 0 |
|  |  |  |  |  |  |  |  | Ha’asini | 14 | 0 | 0 |
|  |  |  |  |  |  |  |  | Holonga | 10 | 0 | 0 |
|  |  |  |  |  |  |  |  | Tatakamotonga | 26 | 0 | 0 |
|  |  |  |  |  |  |  |  | Nakolo | 7 | 0 | 0 |
|  |  |  |  |  |  |  | Vaini | Folaha | 26 | 0 | 0 |
|  |  |  |  |  |  |  |  | Ha’ateiho | 42 | 0 | 0 |
|  |  |  |  |  |  |  |  | Longoteme | 15 | 0 | 0 |
|  |  |  |  |  |  |  |  | Vaini | 78 | 0 | 0 |
|  |  |  |  |  |  |  |  | Veitongo | 31 | 0 | 0 |
|  |  |  |  |  |  |  |  | Tokomololo | 92 | 0 | 0 |
|  |  |  |  |  |  |  |  | Pea | 46 | 0 | 0 |
|  |  |  |  |  |  |  |  | Malapo | 10 | 0 | 0 |
|  |  |  |  |  |  | Ha’apai | Foa | Faleloa | 13 | 0 | 0 |
|  |  |  |  |  |  |  |  | Fotua | 29 | 0 | 0 |
|  |  |  |  |  |  |  | Ha’ano | Fakakai | 8 | 0 | 0 |
|  |  |  |  |  |  |  |  | Ha’ano | 3 | 0 | 0 |
|  |  |  |  |  |  |  |  | Mo’unga’one | 2 | 0 | 0 |
|  |  |  |  |  |  |  | Lulunga | Tungua | 7 | 0 | 0 |
|  |  |  |  |  |  |  |  | Ha'afeva | 7 | 0 | 0 |
|  |  |  |  |  |  |  |  | Fotouha'a | 5 | 0 | 0 |
|  |  |  |  |  |  |  |  | Kotu | 11 | 0 | 0 |
|  |  |  |  |  |  |  |  | Matuku | 5 | 0 | 0 |
|  |  |  |  |  |  |  |  | 'O'ua | 5 | 0 | 0 |
|  |  |  |  |  |  |  | Nomuka | Nomuka | 17 | 1 (5.9) | 0 |
|  |  |  |  |  |  |  |  | Mango | 1 | 0 | 0 |
|  |  |  |  |  |  |  |  | Fonoiufa | 3 | 0 | 0 |
|  |  |  |  |  |  |  | Pangai | Koulo | 17 | 0 | 0 |
|  |  |  |  |  |  |  |  | Hihifo | 25 | 0 | 0 |
|  |  |  |  |  |  |  |  | Pangai | 56 | 0 | 0 |
|  |  |  |  |  |  |  | Uiha | Felemea | 11 | 1 (9.0) | 0 |
|  |  |  |  |  |  |  |  | Lofanga | 2 | 0 | 0 |
|  |  |  |  |  |  | ‘Eua | ‘Eua Fo’ou | Angaha | 62 | 0 | 0 |
|  |  |  |  |  |  |  | ‘Eua Motu’a | Ha’atu’a | 35 | 0 | 0 |
|  |  |  |  |  |  |  |  | Tufuvai | 10 | 0 | 0 |
|  |  |  |  |  |  |  |  | ‘Ohonua | 57 | 0 | 0 |
|  |  |  |  |  |  |  |  | Houma | 4 | 0 | 0 |
|  |  |  |  |  |  | Vava’u | Hahake | Koloa | 20 | 0 | 0 |
|  |  |  |  |  |  |  |  | Houma | 8 | 0 | 0 |
|  |  |  |  |  |  |  |  | Ha’alaufuli | 17 | 0 | 0 |
|  |  |  |  |  |  |  |  | Tu’anekivale | 18 | 0 | 0 |
|  |  |  |  |  |  |  | Hihifo | Tu’anuku | 8 | 0 | 0 |
|  |  |  |  |  |  |  |  | Taoa | 9 | 0 | 0 |
|  |  |  |  |  |  |  |  | Longomapu | 18 | 0 | 0 |
|  |  |  |  |  |  |  |  | Tefisi | 19 | 0 | 0 |
|  |  |  |  |  |  |  | Leimatu’a | Feletoa | 28 | 0 | 0 |
|  |  |  |  |  |  |  |  | Holonga | 9 | 0 | 0 |
|  |  |  |  |  |  |  |  | Leimatu'a | 25 | 0 | 0 |
|  |  |  |  |  |  |  | Motu | Falevai | 2 | 0 | 0 |
|  |  |  |  |  |  |  |  | Hunga | 8 | 0 | 0 |
|  |  |  |  |  |  |  |  | Kapa | 1 | 0 | 0 |
|  |  |  |  |  |  |  |  | Lape | 3 | 0 | 0 |
|  |  |  |  |  |  |  |  | Matamaka | 2 | 0 | 0 |
|  |  |  |  |  |  |  |  | Taunga | 3 | 0 | 0 |
|  |  |  |  |  |  |  |  | 'Otea | 1 | 0 | 0 |
|  |  |  |  |  |  |  | Neiafu | Toula | 16 | 0 | 0 |
|  |  |  |  |  |  |  |  | Ofu | 3 | 0 | 0 |
|  |  |  |  |  |  |  |  | Olo'ua | 2 | 0 | 0 |
|  |  |  |  |  |  |  |  | Neiafu | 92 | 0 | 0 |
|  |  |  |  |  |  |  |  | Makave | 23 | 0 | 0 |
|  |  |  |  |  |  |  | Pangaimotu | 'Utulei | 3 | 0 | 0 |
|  |  |  |  |  |  |  |  | 'Untungake | 6 | 0 | 0 |
|  |  |  |  |  |  |  |  | Pangaimotu | 21 | 0 | 0 |
|  |  |  |  |  |  |  | NS | NS | 21 | 0 | 0 |
|  |  |  |  |  |  |  | NS | NS | 10 | 0 | 0 |
|  |  |  |  |  |  | Ongo Niua | Niuafo’ou | Sapaata | 11 | 0 | 0 |
|  |  |  |  |  |  |  |  | Tongamama’o | 3 | 0 | 0 |
|  |  |  |  |  |  |  | Niuatoputpau | Falehau | 8 | 1 (12.5) | 0 |
|  |  |  |  |  |  |  |  | Hihifo | 14 | 1 (7.1) | 0 |
| TAS3 / Post-MDA Surveillance (2015) |  |  | School-based survey of all Grade 1 children (approximately 6 years old). | 2,806 | 1 (0.04) | Tongatapu |  |  | 2022 | 0 | NT |
|  |  |  |  |  |  | Vava'u |  |  | 386 | 0 | NT |
|  |  |  |  |  |  | Ha'apai |  |  | 213 | 0 | NT |
|  |  |  |  |  |  | ‘Eua |  |  | 147 | 0 | NT |
|  |  |  |  |  |  | Ongo Niua |  |  | 20 | 1 (5.0%) | NT |
| Validation of Elimination of Lymphatic Filariasis as a Public Health Problem (2017) | | | | | | | | | | | |
