# Supplementary material for: Operational research to inform post-validation surveillance of lymphatic filariasis in Tonga study protocol: History of lymphatic filariasis elimination, rational, objectives, and design
Source: PLoS One. 2024 Aug 20;19(8):e0307331. doi: 10.1371/journal.pone.0307331 (PMC11335152; doi:10.1371/journal.pone.0307331)
Supplement: Attachment — Submitted filename: Response to requested edits.docx [file pone.0307331.s002.docx]

**1. Please complete all items on the Human Participants Research Checklist:**

Please find a completed Human Participants Research Checklist uploaded as an ‘Other’ file.

A section regarding the date of participant recruitment has been added in lines 274-276.

**2.Please provide additional details regarding participant consent:**

Signed consent will be obtained from participants aged ≥18 years, or from parents/guardians for participants aged <18 years. Further elaboration on the type of informed consent has been included in the section **Eligibility criteria and consent process** from line 210.

**3. We note you did not include a copy of the original, (signed or stamped) dated IRB/research ethics committee approval letter or waiver for all study sites as an ‘Other’ file:**

Ethical approval from the Tonga NHERC and UQ HREC have been uploaded as ‘Other’ files.

**4. We note you did not include a letter from an external funder confirming the funding award you received as an ‘Other’ file:**

Confirmation of funding has been uploaded as an ‘Other’ file.
